# Socio-Economic Impact of the Covid-19 Pandemic in the U.S.

**DOI:** 10.3390/e23060673

**Published:** 2021-05-27

**Authors:** Jonathan Barlow, Irena Vodenska

**Affiliations:** 1 Department of Physics, Graduate School of Arts and Sciences, Boston University, Boston, MA 02215, USA; vodenska@bu.edu; 2 Laboratory for Interdisciplinary Finance and Economics (LIFE) Research, Metropolitan College, Boston University, Boston, MA 02215, USA; 3 Department of Administrative Sciences, Metropolitan College, Boston University, Boston, MA 02215, USA; 4 Global Development Policy Center, Boston University, Boston, MA 02215, USA

**Keywords:** Covid-19, complex networks, resilience

## Abstract

This paper proposes a dynamic cascade model to investigate the systemic risk posed by sector-level industries within the U.S. inter-industry network. We then use this model to study the effect of the disruptions presented by Covid-19 on the U.S. economy. We construct a weighted digraph G = (V,E,W) using the industry-by-industry total requirements table for 2018, provided by the Bureau of Economic Analysis (BEA). We impose an initial shock that disrupts the production capacity of one or more industries, and we calculate the propagation of production shortages with a modified Cobb–Douglas production function. For the Covid-19 case, we model the initial shock based on the loss of labor between March and April 2020 as reported by the Bureau of Labor Statistics (BLS). The industries within the network are assigned a resilience that determines the ability of an industry to absorb input losses, such that if the rate of input loss exceeds the resilience, the industry fails, and its outputs go to zero. We observed a critical resilience, such that, below this critical value, the network experienced a catastrophic cascade resulting in total network collapse. Lastly, we model the economic recovery from June 2020 through March 2021 using BLS data.

## 1. Introduction

A central problem in macroeconomic research is determining the origins of large-scale fluctuations in aggregate indicators, such as the total factor productivity, GDP, inflation, and employment. A variety of ideas have been proposed to explain these fluctuations, such as the optimal capital accumulation in response to business cycles (Kydland and Prescott [1]), or the result of monetary policies (Farhi and Werning [2]). A growing branch of research has focused on the propagation of shocks based on the implicit connections of economic institutions, based on ideas drawn from network science. While the network approach today is widely known and frequently employed, it has experienced criticism, notably by Lucas [3].

In his 1977 paper, Lucas presented the diversification argument where he argued that as the representation of an economy becomes increasingly disaggregated, independent shocks will average out, and the aggregate volatility will converge rapidly. The level of disaggregation is considered as the number of sectors, *n*, that comprise an economy. The manufacturing industry, for example, may be divided into sub-sectors, such as paper, textiles, rubber, and electrical components. These sub-sectors may be further divided, all the way down to the level of individual firms. The diversification argument is essentially a statement of the Law of Large Numbers (LLN): as the sample size (industrial disaggregation) grows, the average of all independent, identically distributed shocks approaches the expected value.

However, as shown by Acemoglu et al. [4], the diversification argument neglects the interdependencies present between industries, in essence assuming that each industry is its sole supplier. As an extreme example, Acemoglu et al. considered a network structure in which one industry is the sole supplier to all other industries. Here, even as *n* grows, shocks to the supplying industry propagate through the network, creating significant aggregate fluctuations. On the other hand, homogeneous networks in which each industry contributes equally to all others follow an LLN type argument.

For network structures that lie between these two extremes, e.g., the U.S. economy, they found that the aggregate volatility did converge for increasing *n*, albeit at a slower rate than suggested by Lucas. Gabaix [5] formulated a similar rebuttal: when the firm size distribution displayed heavy tails, idiosyncratic shocks to larger firms could not be averaged away by shocks to smaller ones. Thus, it is evident that, while the level of disaggregation is a factor, the network structure plays a crucial role in generating macroeconomic volatility.

In this paper, we investigate the systemic risk present in the U.S. industrial network and the economic impact of the Covid-19 pandemic. To model the network, we construct a weighted digraph G(V,E,W) based on input–output tables, where the vertices $V_{i}$ represent sector level industries, edges $E_{ij}$ represent a directed commodity flow from industry $V_{i}$ to $V_{j}$, and the edge weight $W_{ij}$ captures the value of the commodity flow. The production of an industry relies on the supply of its upstream industrial products. If an upstream industry fails to produce normally, the reliant industry’s production capacity will be reduced, further propagating the initial production shortage in the network.

We model the production of each industry via a modified Cobb–Douglas function, where an industry’s outputs rely on their productivity, labor input, and industrial inputs from upstream suppliers. The propagation of production shortages is evaluated with a dynamic cascade model, in which the production decreases according to the rate of loss in the inputs over subsequent periods, scaled by the parameters of the Cobb–Douglas function. The dynamic model includes the possibility of industry failure via a threshold function dependent on the resilience (or ability to absorb losses), which is assumed to be uniform across all industries.

To study the systemic risk present in the network, we first simulate an initial general production shock to a particular industry, and find that there exists a critical resilience below which the network suffers a catastrophic cascade resulting in total failure of the network. These simulations are performed for each industry, for varying levels of initial disruption. We then compare the response of the network under each scenario and identify the systemically important industries.

Using the methodology for single industry shocks, we model the disruption of the Covid-19 pandemic by the observed unemployment spike between March and April 2020. We identify the critical resilience $r_{c}$ for the Covid-19 shock, above which the network experiences varying levels of reduced production, and below which the network collapses. Our model is generalizable, such that it may serve as a valuable tool for estimating the economic impact of a wide variety of disruption scenarios, whether it be an endogenous economic disruption, public health crisis, or natural disaster catastrophe. Additionally, the model can be applied at various scales; locally, nationally, or globally.

### Literature Review

Our work intersects with two main research areas, systemic risk modeling and the socioeconomic effects of the Covid-19 pandemic.

Economic networks have been used to study the periodic dynamics of business cycles and model systemic risk, i.e., to assess the potential for failure of the entire economic system or market. The process by which individual entities in these systems fail is known as cascading failure, akin to the domino effect, where the failure of one entity triggers an avalanche of successive failures. For example, during the global financial crisis (GFC) of 2007–2008, the failure of one or more banks contributed to systemic risk propagation throughout the global financial network.

Researchers have used bipartite network models to study systemic risk propagation in different economic scenarios, including the Japanese banking crisis in the 1990s (Sakamoto and Vodenska [6]), the 2007–2008 U.S. financial system distress (Huang et al. [7], Garas et al. [8]), and the 2011 Eurozone sovereign debt crisis (Vodenska et al. [9]). To identify systemically important banks in the U.S., Battiston et al. [10] introduced a novel centrality measure known as DebtRank. Its contribution extends the typical network centrality idea to include the effects of a distressed node on the network.

Similarly, Vodenska et al. [11] introduced a metric called BankRank for dynamic bipartite networks, and applied it to the 2011 Eurozone sovereign debt crisis. DebtRank and BankRank are inspired by another centrality measure known as PageRank (Page et al. [12]), which sought to organize search engine results, and has since been adopted by Google. We add to this literature by constructing a new systemic risk ranking system, one based on distressed nodes in a weighted digraph, and apply it to the U.S. industrial network.

As the Covid-19 pandemic spread globally, the connections present in regional to global economies were stressed in unprecedented ways. Public health concerns mandated shutdowns and shelter-in-place orders, triggering the highest unemployment rates in large economies since the Great Depression. Moreover, personal consumption habits were drastically re-evaluated, shifting away from social sectors, such as leisure and travel, due to fears of infection. These multi-faceted effects passed through different economic sectors, presenting a unique challenge in describing aggregate behavior.

Examples of the concerns raised by this pandemic include whether initial supply shocks can lead to deficits in demand (Brinca et al. [13]), how the magnitudes of supply and demand shocks compare across industries (Guerrieri et al. [14]), and what socioeconomic implications of a global pandemic might persist in the future (Nicola et al. [15]). Other research focused on the impact to employment caused by the health crisis; Lee et al. [16] analyzed the impact on employment rates and their subsequent recoveries across demographics, Blustein et al. [17] presented avenues for research on strategies for dealing with the large unemployment rates, and identifying vulnerable workers, Milani [18] found that the response in unemployment varied across countries and that the U.S. employment rate was particularly susceptible to health shocks, and Farrell et al. [19] investigated the relationship between unemployment insurance and aggregate consumption.

Barrot et al. [20] used a general equilibrium model with Cobb–Douglas technologies to determine the economic impact of social distancing and the resulting labor reduction in France, Carvalho and Tahbaz-Salehi [21] presented a literature review of these production network models. Luo et al. [22] used a static network model with constant elasticity of substitution (CES) production function to determine the economic damage of the stay-at-home orders in the U.S.

Makridis and Hartley [23] estimated U.S. GDP reduction per month of economic shutdown, by assuming that businesses stayed operational in proportion to their degree of digitalization. We contribute to this research area by estimating the U.S. GDP reduction due to Covid-19, utilizing the industry level unemployment and productivity data as inputs to our proposed dynamic model.

## 2. Materials and Methods

To characterize the interrelationships between sectors of an economy, Wassily Leontief formulated the well-known input–output (IO) model, the first matrix representation of an economy. This model states that the total output of an industry consists of the output delivered directly to the final consumers, plus the output used intermediately by other industries as inputs to make their own products. The total industrial output is captured by the following equation:

(1) $$x_{i}=\sum_{j}a_{ij}x_{j}+d_{i}$$

where the *i*’th industry’s total output, $x_{i}$, depends on the final demand in that industry, $d_{i}$, as well as the intermediate input required from each industry, $a_{ij}x_{j}$. The coefficients $a_{ij}$ captures the input share, i.e., how much does industry *i* need to produce so that industry *j* can produce one unit of product. These coefficients $a_{ij}$ form the direct requirements of matrix *A*:

(2) $$A=\left[\begin{array}{ccc} a_{11} & \cdots & a_{1n}\\ \vdots & \ddots & \vdots \\ a_{n1} & \cdots & a_{nn} \end{array}\right]$$

Written in this matrix form, the output and demand become vectors containing the entries for each industry, x=Ax+d. Solving for the output vector x yields $x={\left(1-A\right)}^{-1}d$, where ${\left(1-A\right)}^{-1}$ is known as the Leontief inverse, providing us with a straightforward method to determine how the levels of final demand affect the output. More commonly, this method is used to determine the necessary changes in output to compensate for changes in the final demand, $\Delta x={\left(1-A\right)}^{-1}\Delta d$.

### 2.1. Input–Output Model

To illustrate this model, we construct an economy comprised of two sectors, goods (1) and services (2). The input–output model will have the following form, also known as an impact model:

(3) $$\left[\begin{array}{c} \Delta x_{1}\\ \Delta x_{2} \end{array}\right]=\left[\begin{array}{cc} 1.5 & 0.2\\ 0.4 & 1.2 \end{array}\right]\left[\begin{array}{c} \Delta d_{1}\\ \Delta d_{2} \end{array}\right]$$

To study the network effect, we assume that the change in demand for goods is increased by 10%, while there is no change in the demand for services. For the output vector, we find that $\Delta x_{1}=15\%$, and $\Delta x_{2}=4\%$. Even though there was no change in demand for services, we observe the increased output of services to satisfy the increased demand for goods. This idea can be expressed by decomposing the input–output equation as follows:

(4) $$\Delta x_{i}=\Delta d_{i}+\sum_{j}\left(l_{ij}-\delta_{ij}\right)\Delta d_{j}$$

The values of $l_{ij}$ are the entries of the Leontief inverse, and $\delta_{ij}$ is the Kronecker delta function (equal to 1 if i=j). The first term represents the immediate impact of a change in demand for industry *i* on the output of industry *i*. The second term conveys the network effect, where changes in demand of other industries also impact the output of industry *i*. Hence, the elements of the Leontief inverse capture the network connections present in the economic structure.

The economic impact model described in Equation (1) is used to estimate the change in economic activity in a specified region due to an external influence, such as a new business, policy, technology, project, or organization. The impact may be described in terms of the change in the total output of an economy, labor income, or employment. A commonly used model is the Regional Input–Output Modeling System (RIMS II) provided by the BEA, which uses the elements of the Leontief inverse to calculate the relevant multipliers depending on the nature of the impact. These types of I.O. models are referred to as “fixed-price” models, meaning that businesses may use as many inputs as necessary without facing changes in price.

The second class of economic impact model is known as a general equilibrium model, in which there is no assumption of fixed prices. These equilibrium models forecast the changes in economic activity levels estimated by I.O. models, including the impact of price and wage changes. Such a general equilibrium model is used in real business-cycle theory, which models a perfectly competitive economy in which firms and households seek to maximize their utility subject to budget constraints. Models of real business cycles today, such as those proposed by Acemoglu et al. [24] and Carvalho [25] were built on the original theory of Plosser and Long [26] by incorporating the inter-industry networks used in the simpler IO models.

Our model utilizes ideas present in both I.O. impact models and real business-cycle theory. Namely, we use the Leontief inverse to construct our inter-industry network under the assumption that industries follow a Cobb–Douglas production function. Our model does not use the elements of the Leontief inverse as static multipliers to calculate changes in economic activity, but rather, the Leontief inverse elements are allowed to fluctuate to represent changes in the output capacity of industries subject to disruption. This allows us to investigate the dynamics of damage spreading in the network over time, as opposed to the static nature of an impact model. Additionally, the inclusion of a resilience parameter controlling industry failure allows our model to make estimates of systemic risk, an aspect that is not present in impact models or general equilibrium models.

To construct the industrial network, we use the industry-by-industry total requirements table provided by the BEA for 2018, with the disaggregation level n=15. The network represents a weighted digraph, with industries as nodes and production relationships as links. An entry $M_{ij}$ in the matrix represents the production required by industry *i* to satisfy industry *j*’s output. At this sector level, the network is complete; however, some weights are nearly zero.

As a basis for estimating the loss in output due to an initial shock, we build on the Cobb–Douglas production function for multiple goods as follows:

(5) $$Y_{i}=A_{i}L_{i}^{\alpha }\prod_{j}x_{ij}^{\gamma (\omega_{ij})}$$

where the output of an industry $Y_{i}$ depends on the productivity ($A_{i}$), labor input ($L_{i}$), and intermediate inputs from the *j*’th industry, ($x_{ij}$). The exponents α and γ are known as the elasticities of labor and input, respectively, and capture the weight with which each factor affects the output. The term $\omega_{ij}$ is the share of input *j* in industry *i*’s total inputs. In this formulation, the production function exhibits constant returns to scale, provided $\sum_{j}\omega_{ij}=1$ and α+γ=1. This guarantees that, if labor and all intermediate inputs double, for example, then the output will also double.

An initial shock may be modeled by a decrease in productivity or labor loss that reduces the total output of the shocked industry. As this initial shock reduces the output, and each downstream industry will, in turn, be affected by a loss of inputs, which further reduces the output. The propagation of damage depends on the type of shock as well as the network structure. Further, the spread of damage may be compounded by the failure, or exit, of industries in the network. In the following section, we describe the propagation method and a failure condition.

### 2.2. Dynamic Cascade Model

We propose a dynamic cascade model that estimates the change in total production over time due to an initial negative shock in production capacity, by either a reduction in productivity or labor supply. The propagation of damage and failure conditions depend on the rate of change in the total inputs over adjacent periods. This dynamic condition may be described as allowing industries to compensate for reduced inputs, as long as the input change does not exceed the failure threshold. The dynamic model runs for t∈[0,T] time periods, with a negative shock applied at t=0. In this model, we modify the Cobb–Douglas production function of Equation (5) to allow an industry to fail without resulting in an output of zero for the entire system.

We reformulate Equation (5) as follows:

(6) $$Y_{i}=A_{i}L_{i}^{\alpha }{\left(\sum_{j}x_{ij}\right)}^{\gamma }$$

Hence, an industry is still able to produce a fraction of its original output even though one or more of its input suppliers have exited the network. An initial labor shock has the following effect:

(7) $$Y_{i}^{1}={\left(S_{i}\right)}^{\alpha }\times Y_{i}^{0}\;;\;\mathrm{where}\;S_{i}=\frac{L_{i}^{1}}{L_{i}^{0}}$$

with the superscript denoting the time period *t*. Equation (7) states that the output of industry *i* at t=0, $Y_{i}^{0}$, is reduced to a fraction ${\left(S_{i}\right)}^{\alpha }$ of itself at t=1, $Y_{i}^{1}$, where $S_{i}$ is the ratio of labor inputs in industry *i* between the two consecutive periods, and α is the elasticity of labor (labor share). For instance, if α=0.7 and the initial reduction in labor in industry *i* is 50%, the output of industry *i* at t=1 will be 62% ($=0.5^{0.7}\times 100\%$) of its original output at t=0. Importantly, since the reduction is uniform across all outputs, the total output is reduced by the same fraction. The damage due to loss of inputs is spread during subsequent periods as follows:

(8) $$Y_{i}^{t+1}={\left(p_{i}^{t}\right)}^{\gamma }\times Y_{i}^{t}\;;\;\mathrm{where}\;p_{i}^{t}=\frac{\sum_{j}x_{ij}^{t}}{\sum_{j}x_{ij}^{t-1}}$$

Equation (8) has the same structure as Equation (7), where the output of industry *i* in period *t* is reduced to a fraction ${\left(p_{i}^{t}\right)}^{\gamma }$ of itself in period t+1. The value of $p_{i}^{t}$ is the ratio of the total inputs in period *t* over the total inputs in the previous period (t−1), and γ is the elasticity of the inputs. The outputs of industry *i* are reduced uniformly by the value ${\left(p_{i}^{t}\right)}^{\gamma }$, and the propagation term $p_{i}^{t}$ depends on the rate of change of inputs in successive periods.

Each industry has a uniform resilience level *r*, such that if $p_{i}^{t}>r$ industry *i* fails, i.e., its outputs go to zero. In other words, the resilience of an industry determines the sustainable marginal reduction in inputs. For example, if r=0.2, industries in the network can withstand up to a 20% reduction in inputs in any period without failing. We run the model simulation for various values of r∈[0,1], allowing us to determine the critical resilience $r_{c}$. We define the critical resilience $r_{c}$ of the network such that, for all $r\leq r_{c}$, the cascade results in a total collapse (all industries fail).

To illustrate the model, we construct an economy comprised of four sectors (A, B, C, and D). For simplicity, all sectors contribute 1 unit of intermediate input to every other sector in the network. In this economy, the industries’ outputs are given along the rows, and the industries’ inputs are given along the corresponding columns. We chose the resilience (*r*) for each industry to be 0.2, and industry A’s outputs were initially shocked by 50%. These parameter values were chosen to provide a straightforward example of the model.

(9) $$\begin{array}{cc} \begin{array}{cccc} A_{in} & B_{in} & C_{in} & D_{in} \end{array} & \\ \left(\begin{array}{cccc} \;\;1\;\; & \;\;1\;\; & \;\;1\;\; & \;\;1\;\;\\ 1 & 1 & 1 & 1\\ 1 & 1 & 1 & 1\\ 1 & 1 & 1 & 1 \end{array}\right) & \begin{array}{c} A_{out}\\ B_{out}\\ C_{out}\\ D_{out} \end{array} \end{array}\;\;\underset{\mathrm{shock}}{\to }\;\begin{array}{cc} \begin{array}{cccc} A_{in} & B_{in} & C_{in} & D_{in} \end{array} & \\ \left(\begin{array}{cccc} \;0.5\; & \;0.5\; & \;0.5\; & \;0.5\;\\ 1 & 1 & 1 & 1\\ 1 & 1 & 1 & 1\\ 1 & 1 & 1 & 1 \end{array}\right) & \begin{array}{c} A_{out}\\ B_{out}\\ C_{out}\\ D_{out} \end{array} \end{array}$$

After the initial shock, we check if this will cause any industries to fail by measuring the rate of input loss. Each column sum in the new matrix is 12.5% (1−3.5/4) less than the previous sum. This loss of input is within the 20% resilience, and thus no industry fails due to the initial shock. The next step is to calculate the first iteration of damage spreading. The resulting loss of output affects all industries, equally in this case, related to the 12.5% loss from the shock, modified by the elasticity of the inputs (typically γ=0.3). Therefore, ${\left(1-0.125\right)}^{0.3}=0.961$, meaning that each industry now reduces their output uniformly by about 4%.

(10) $$\begin{array}{cc} \begin{array}{cccc} A_{in} & B_{in} & C_{in} & D_{in} \end{array} & \\ \left(\begin{array}{cccc} \;0.5\; & \;0.5\; & \;0.5\; & \;0.5\;\\ 1 & 1 & 1 & 1\\ 1 & 1 & 1 & 1\\ 1 & 1 & 1 & 1 \end{array}\right) & \begin{array}{c} A_{out}\\ B_{out}\\ C_{out}\\ D_{out} \end{array} \end{array}\;\;\overset{\mathrm{reduce}}{\underset{4\%}{\to }}\;\begin{array}{cc} \begin{array}{cccc} A_{in} & B_{in} & C_{in} & D_{in} \end{array} & \\ \left(\begin{array}{cccc} 0.48 & 0.48 & 0.48 & 0.48\\ 0.96 & 0.96 & 0.96 & 0.96\\ 0.96 & 0.96 & 0.96 & 0.96\\ 0.96 & 0.96 & 0.96 & 0.96 \end{array}\right) & \begin{array}{c} A_{out}\\ B_{out}\\ C_{out}\\ D_{out} \end{array} \end{array}$$

This process of checking for industry failure, then propagating damage repeats until the maximum period *T* is reached, or the network collapses. In the case considered here, the cascade rapidly diminishes due to the survival of all industries, with the subsequent reduction in the output being just over 1%. If the initial shock were >80%, it would have exceeded the resilience of 0.2, and each industry would have failed simultaneously. In the example described in Equation (10), the relative reduction at each step of the cascade is identical for each industry due to the uniformity at the level of inputs. In the U.S. industrial network, which we study, the propagation of damage is more complex due to the variations in production relationships among industries. A visual representation of the weighted links for the U.S. inter-industry network can be found in Figure A3 of Appendix A.

## 3. Results

Using the model described in Section 2.2, we study the effect on the U.S. economy given two different types of shocks, a single-industry shock, and a Covid-19-specific shock. We analyze the dynamics of the network under each stress scenario to understand the impact of the different origins of the shock on the network’s final state. We identify the resilience of the economic network and the industries with the most significant contribution to the systemic risk, under different types of perturbations. Additionally, we assess the possibility and level of recovery given the nature and the severity of the shocks.

### 3.1. Single Industry Shocks

**Figure 1:**
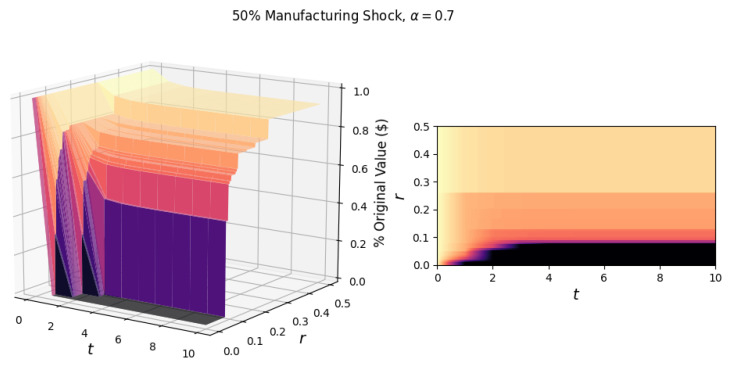
The surface (left) shows the response of the network to a general 50% manufacturing shock for t∈[0,10] and r∈[0,0.5]. On the right is the projection of the surface in the *r*-*t* plane. The critical resilience of the network due to this shock is $r_{c}=0.080$. The elasticities of labor and industrial inputs (α, γ) are 0.7 and 0.3, respectively.

To begin our analysis of shock propagation in the U.S. industrial network, we first look at the simulated effect of shocking a single industry. Unless stated otherwise, we maintain the notion of constant returns to scale, with the labor and input elasticity being 0.7 and 0.3, respectively. To visualize the response of the network to a single industry shock, in Figure 1, we plot the fraction of the original value (in dollars) in the network in each period *t*, for various values of network resilience *r*. While an entry $x_{ij}$ in the total requirements matrix is a relative value, we scale these values by the total value added (for the year 2018, reported by BEA) in each industry to obtain a dollar estimate of the output.

As an illustrative example of a single industry shock scenario, we show the response of the network to a general shock, which reduces the output of the manufacturing industry by 50% (Figure 1). This shock scenario could be realized by reducing the productivity by 20%, labor by 40%, and inputs by 31%. (For a manufacturing shock of 50%, a corresponding reduction in factors could be: $0.5=\left(1-0.2\right){\left(1-0.4\right)}^{0.7}{\left(1-0.31\right)}^{0.3}$, which comes from the Cobb–Douglas production function $Y_{i}=A_{i}L_{i}^{\alpha }{\left(\sum_{j}x_{ij}\right)}^{\gamma }$).

In Figure 1, we observe diminishing cascades due to the initial shock as portions of the surface plateau after time *t*. Following the initial shock, the propagation of damage occurs due to the loss of industrial inputs. The magnitude of the loss is determined by the elasticity of the input γ. Another factor of input loss is the failure of industries based on different network resilience levels. For scenarios with a low network resilience, the 50% manufacturing shock is sufficient to cause a cascade of failures resulting in total network collapse.

The observed critical resilience, $r_{c}$, for the manufacturing shock in Figure 1 is 8.0%, i.e., if the industries in the network can not sustain an 8.0% input loss over a single period, the network will experience a catastrophic collapse. Additionally, the surfaces illustrate the failures of industries, with the highest surface (most negligible value lost) corresponding to scenarios in which no industries fail. As we move to lower values of *r*, we step down the surface. These steps correspond to the failure of one or more industries, the first and largest “step” being the failure of the manufacturing industry.

**Figure 2:**
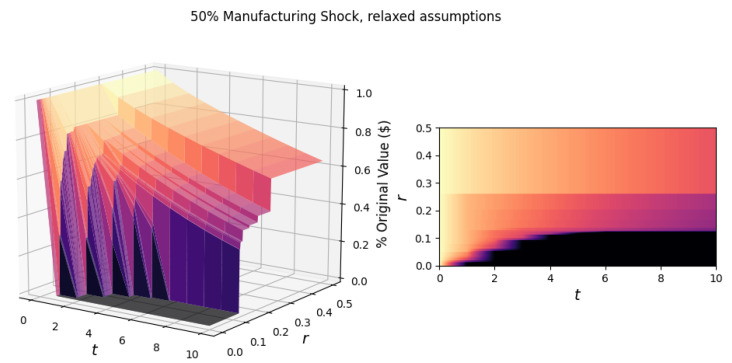
The surface (left) shows the response of the network to a general 50% manufacturing shock, for t∈[0,10] and r∈[0,0.5]. On the right is the projection of the surface in the *r*-*t* plane. The input elasticity γ for each industry is equal to 1. The critical resilience of the network to this shock is $r_{c}=0.126$.

To explore a broader range of cascade scenarios, we relax the constant returns to scale assumption, setting the input elasticity γ=1, which means that the percentage change in output for each industry is identical to the percentage change in input in the previous period. The labor input elasticity remains α=0.7. We then repeat the simulation, subjecting the manufacturing industry to a general shock that decreases its output by 50%, as shown in Figure 2. We see that the higher elasticity of input significantly increased the spread of damage for all resilience levels.

For instance, in the region of no industry failure (for network resilience r>0.3), after 10 periods, the network loses almost 40% of its original value. The change in γ also increased the critical resilience, $r_{c}$, of the network from 0.080 to 0.126, increasing the necessary resilience level to survive. For example, if r=0.095, the network will survive in the γ=0.3 case, whereas, in the γ=1 case, the network will collapse. In other words, for the network to remain active, when γ=1, industries need to absorb up to 12.6% reductions in input per period *t*. Additionally, there is a greater variety in the time it takes for the network to collapse between the two cases of γ.

**Figure 3:**
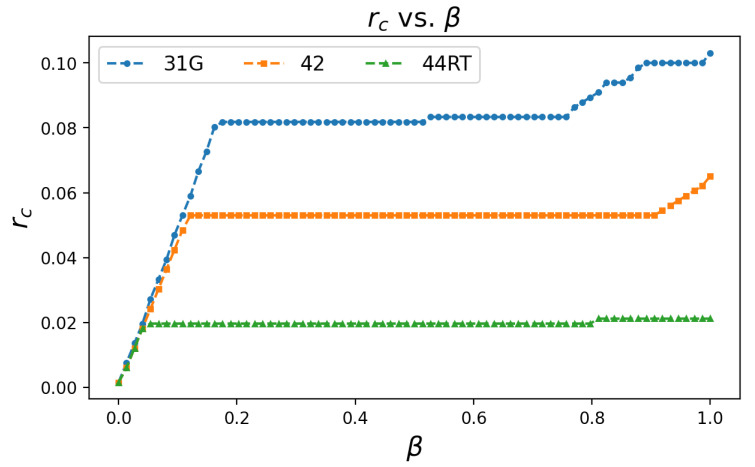
Plot of the critical resilience $r_{c}$ vs. β for single industry shocks on the manufacturing (31G), wholesale trade (42), and retail trade (44RT) industries. This is with input elasticity γ=0.3, and a generalized shock that uniformly reduces outputs at t=0 by the fraction β.

To summarize the results of manufacturing shock scenarios, we show in Figure 3 how the critical resilience varies with the initial general shock magnitude, which we denote β. The magnitude of the shock is between [0,1], where β=0 corresponds to no shock, while β=1 is a total loss in output at t=0. We find a strictly monotonic relationship between critical resilience and shock magnitude. We also observe a large region of β∈[0.16,0.76] that produces nearly the same critical resilience. This result may be due to the localized nature of the initial shock, and the low elasticity of the inputs.

In our model, we assume that reductions in input affect the output in the subsequent time step. In reality, there may be lagged effects that cause damage; however, we do not consider these effects in our model. If the effect of input loss is lagged due to differences in the propagation time of different industries, the relationship between the critical resilience and shock magnitude might not be strictly monotonic.

These lags may shift the time series of failures to allow damage to spread more evenly over time, reducing the value of $p_{i}\left(t\right)$ of Equation (8) for each industry, and thus lowering the critical resilience necessary to avoid failure. On the other hand, lagged effects may cause damage to “pile up” in the same period, increasing the value of $p_{i}\left(t\right)$, which would then raise the critical resilience. Figure 3 also shows the results of the same simulation performed on the Wholesale Trade and Retail Trade industries for comparison.

While two industries may have the same critical resilience for a given shock level, the damage to the entire network may be very different in regions of $r>r_{c}$. Table 1 gives the average values of $r_{c}$ for each single industry $r_{c}$ vs. β simulation. A higher average value of $r_{c}$ means that the network needs to be more resilient to avoid collapse for a specific industry shock scenario. A full set of $r_{c}$ vs. β plots for all 15 sector level industries can be found in Appendix A (Figure A4).

### 3.2. Covid-19 Shock

**Figure 4:**
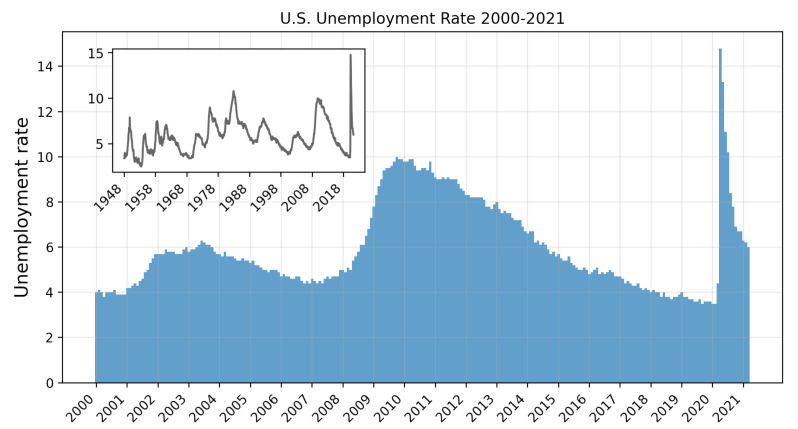
Monthly U.S. unemployment rate (%) from January 2000 to March 2021. The inset plot shows the monthly unemployment rate from January 1948 to March 2021.

In this section, we analyze the shock to the economy due to the global Covid-19 pandemic. This public health crisis has adversely affected the economy to a great extent, with the unemployment rate reaching its highest levels since the post-World War II era. In contrast to the single industry disruption covered in the previous section, the initial shock associated with Covid-19 is broad, affecting every industry with varying severity, and contributing to a significant decrease in economic output. We model the initial shock to the economy by the loss in employment in each industry between March and April 2020 (Table 2). At its peak in April 2020, the unemployment in the U.S. reached 14.7%, exceeding the U.S. unemployment rate of 10% during the Global Financial Crisis of 2008 (Figure 4).

In the Employment Situation News Release for April 2020, the BLS stated that “If the workers who were recorded as employed but absent from work due to ‘other reasons’ (over and above the number absent for other reasons in a typical April) had been classified as unemployed on temporary layoff, the overall unemployment rate would have been almost five percentage points higher than reported (on a not seasonally adjusted basis). However, according to usual practice, the data from the household survey are accepted as recorded. To maintain data integrity, no ad hoc actions are taken to reclassify survey responses.” We use the data as given by the BLS.

For the Covid-19 shock, we maintain constant returns to scale production function, and thus the labor shocks described in Table 2 reduce the output by a smaller fraction given by the labor ratio raised to the elasticity of labor (α=0.7). For example, the Leisure and Hospitality industry experienced the most significant decrease in employment of −47.0%, which, according to our production function, reduces the output to ${\left(1-0.47\right)}^{0.7}$ = 64.1% of its original value.

**Figure 5:**
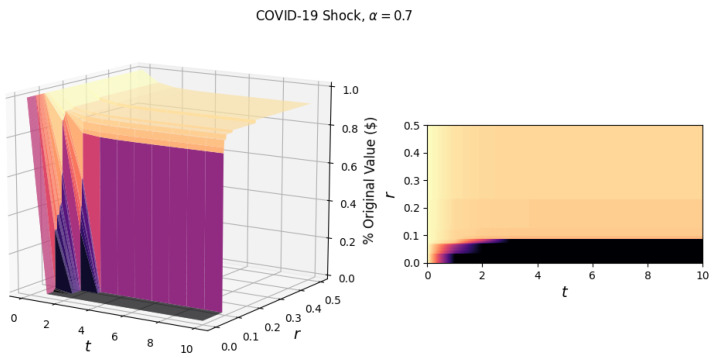
The surface above shows the response of the network to the Covid-19 shock. The resiliency axis, *r*, shows a total network collapse for values of $r\leq r_{c}$, where the critical resilience $r_{c}=0.088$. A closer look at the region $r>r_{c}$ can be found in Appendix A, (Figure A1).

The surface in Figure 5 shows the response of the network to the negative labor shock. While we observe a similar critical resilience $r_{c}$ of 0.088, the cascade dynamics of this multi-industry shock are visibly different from the 50% single industry manufacturing shock (Figure 1). Above the critical resilience $r_{c}$, we see a lower variation in the remaining value in the network. While there are industry failures in this region, the industries that fail are characterized by contributing smaller inputs to the overall network output, as opposed to the large industries that fail in the single industry manufacturing shock.

For the 50% manufacturing shock, just above $r_{c}$, the network ultimately loses 52% of its original value and avoids collapse. Whereas, in the Covid-19 shock, just above $r_{c}$, the network loses 28% of its original value. Even though the two shock scenarios generate similar values of the critical resilience $r_{c}$, the network can absorb more damage in the single industry shock scenario before collapsing. This behavior fits with the model, namely that it is not the total amount of damage that determines failure but rather the rate of damage spreading that does. In the region below the critical resilience, the cascade for the Covid-19 shock is rapid, occurring in the first, second, or third period.

Above the critical resilience, the industries that fail experience the most considerable reduction in employment. The first to fail is the Leisure and Hospitality industry, occurring at a resilience level of about 23%. The failure of Other Services follows with the resilience of around 12%. Between the 12% resilience and the critical resilience, three more industries fail; Retail Trade, Information, and Education and Health Services. The only industry of these five that does not follow from the ranking of initial shocks is Information, which experienced a −9.7% labor shock. Three industries experience more significant initial shocks than Information, yet survived in the region above $r_{c}$: Construction (−13.4%), Manufacturing (−10.3%), and Professional Services (−10.3%).

**Figure 6:**
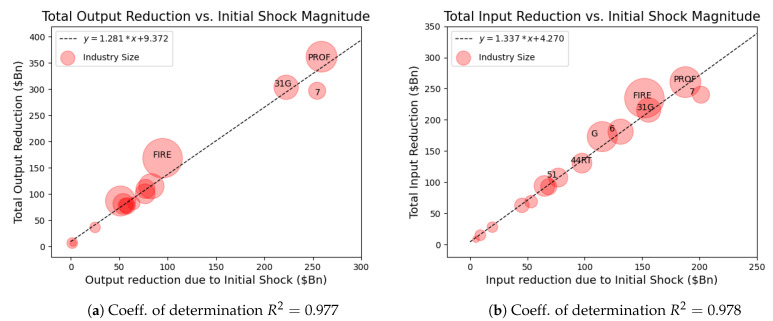
Comparison of the damage experienced by each industry for the region of no industry failure (r>0.23) due to the Covid-19 shock. All axes are in billions of dollars; α=0.7, γ=0.3. (**a**) Plot of the total reduction in output for each industry as a function of the initial shock to input. (**b**) Plot of the total reduction in input for each industry as a function of the initial shock to input.

To study the damage sustained by each industry, we plot the dollar amount of output lost over the entire cascade as a function of the initial shock magnitude for each industry in Figure 6a. We see that the three industries that experienced the most significant initial shock to output (in monetary terms) were the most severely damaged over the entire course of the cascade. The slope of this line is 1.281, which suggests that industry will ultimately incur an additional 28% of damage from the initial Covid-19 labor shock.

An alternate view of the damage spread may be seen by plotting each industry’s total input reduction as a function of the initial shock to inputs (in monetary terms) in Figure 6b. Each industry’s total input reduction is shown to be well explained by that industry’s initial input loss. We see in this figure that Professional Services, Manufacturing, and Leisure and Hospitality are again among the highest damaged industries, joined by Finance, Insurance, and Real Estate.

**Figure A2:**
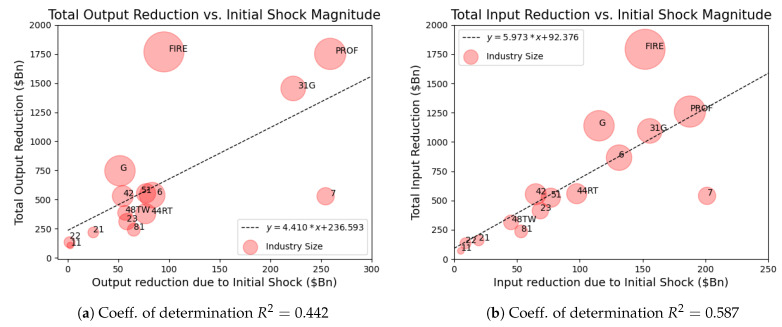
Complementary plots to Figure 6a,b, for the Covid-19 shock with elasticity of inputs γ=1.

**Figure A3:**
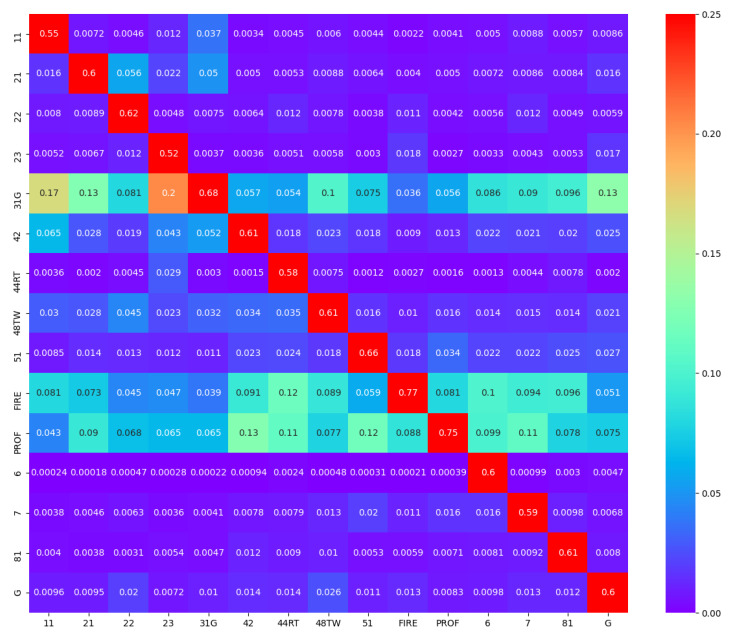
Visualization of the U.S. inter-industry network. Each cell is represented by its weight in the column sum in which it is located. For instance, the first entry of row 31G shows that the output of manufacturing (31G) constitutes 17% of the Agriculture, Forestry, and Fisheries (11) total inputs. We can see that the diagonal entries have the largest weights, implying that an industry’s self-input is the largest component of its total input.

**Figure A4:**
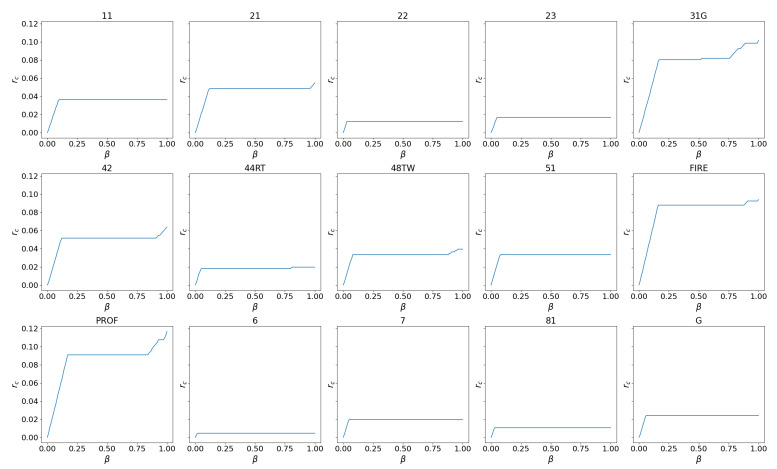
Plots of $r_{c}$ vs. β for single industry shocks of magnitude β, with β=1 being a total loss. The shocked industry is given in the subplot title. The elasticity of the input is set to 1−α=0.3 for all plots.

The linearity of these two plots may be a consequence of the fact that we observe the region of no industry failure (r>0.23), in which cascades diminish rapidly due to the low elasticity of the inputs γ=0.3. If we increase γ, we find that the total value of damage, measured as inputs or outputs, would increase. Additionally, some industries move above the trend line as they experience strong network effects due to their interconnections, as shown in Figure A2 in the Appendix A.

The response of the network to the Covid-19 shock shown in Figure 5 and Figure 6 is solely due to the initial loss in employment and the subsequent loss in inputs. However, in addition to the loss of employment, the pandemic disrupted the processes by which businesses operate. To investigate various productivity effects, we examine how the network responds to a unilateral reduction in productivity (*A*) at a rate *F*.

$$A_{i}\left(t\right)=F^{t}A_{i}\left(0\right)$$

**Figure 7:**
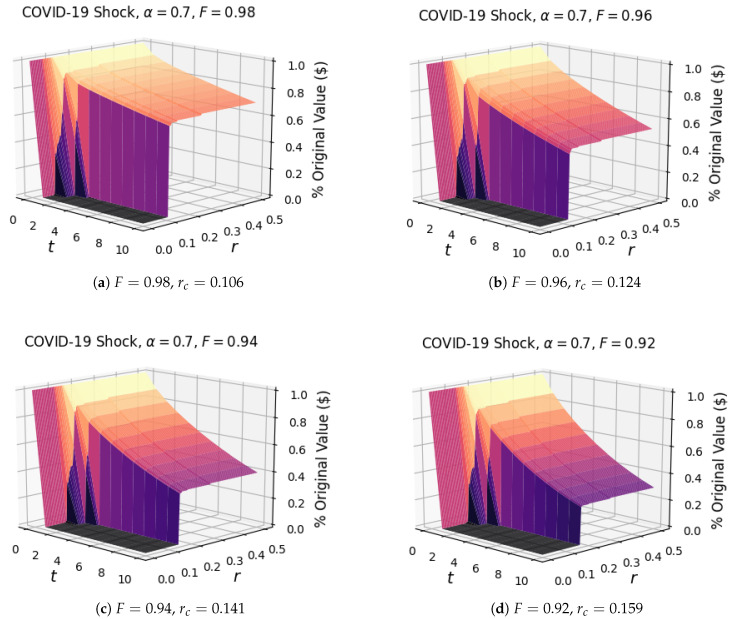
The response of the network to varying exponential reductions in productivity that were initially subject to the Covid-19 employment loss. Subfigures: (**a**) A 2% reduction in productivity in each period, yielding $r_{c}=0.106$; (**b**) a 4% reduction in productivity in each period, yielding $r_{c}=0.124$; (**c**) a 6% reduction in productivity in each period, yielding $r_{c}=0.141$; and (**d**) an 8% reduction in productivity in each period, yielding $r_{c}=0.159$.

Figure 7 shows the network response to (i) the initial labor shock and (ii) an exponential decay in productivity. We see that the value of *F* controlling the decay not only increases the damage propagation for cascades $r>r_{c}$, it also increases the value of $r_{c}$ itself, widening the region of total collapse. For F=0.98, the critical resilience $r_{c}=0.106$, while, for F=0.92, the critical resilience $r_{c}=0.159$. The network is very sensitive to changes in productivity, as compared to decreased inputs or labor loss, because this appears linearly in the production function.

### 3.3. Aftermath and Recovery

In this section, we extend the model to include the entire time series of the monthly employment levels in each industry, from the initial shock in March 2020 to March 2021. In Figure 5, we show that the Covid-19 shock does not lead to a cascade of losses in the region above the critical resilience $r_{c}$. In this region, the network reaches an equilibrium after the shock, where the inputs and outputs are no longer changing appreciably. This result signifies that the recovery will resemble the employment curves (modified by the elasticity of labor). In other words, we expect the return to employment to have a sustainable positive effect on the network.

**Figure 8:**
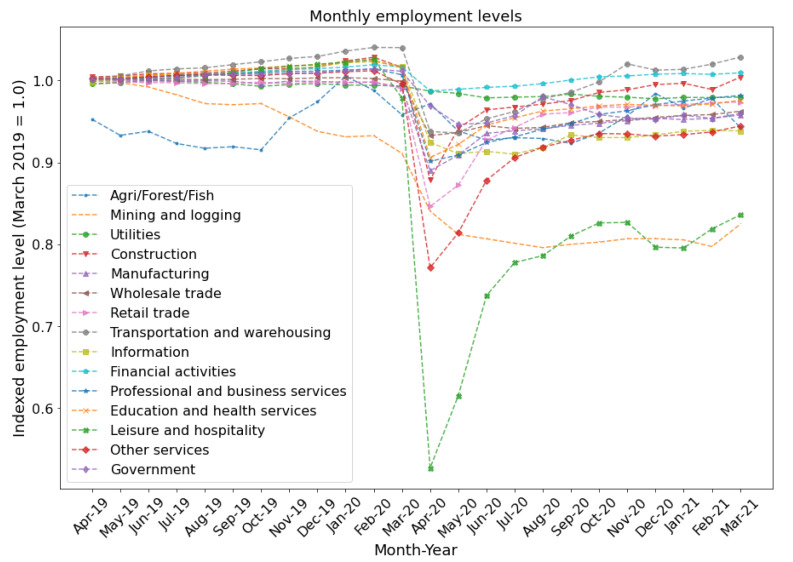
Monthly employment levels in each of the 15 sector level industries over a two year period (April 2019–March 2021), measured as a fraction of the March 2019 employment levels.

In Figure 8, we show the employment trend for each industry from April 2019 to March 2021. We can see the decline in employment between March and April 2020, where the most considerable loss in the employment of −47% occurred in the Leisure and Hospitality industry. We also observe the subsequent employment recovery process, with most industries returning to close to the original employment levels.

We see that in general, each industry (except Mining and Logging) shows some recovery in the year following the shock, with the most rapid changes occurring in May–June 2020 period. Most industries are currently above 90% of their March 2019 levels, while Leisure and Hospitality and Mining and Logging are hovering just above 80% of their March 2019 levels. The industries affected most by Covid-19 (see Table 2 for explicit values) are also those farthest from a full recovery.

In addition to employment data, we analyzed the labor productivity changes in each industry, which is defined as the change in total output per hour worked. We used the BLS productivity data for the Manufacturing industry and the non-farm business sector. We used the non-farm business productivity levels as a proxy for each industry except Manufacturing (31G), Agriculture, Forestry and Fishing (11), and Government (G). The productivity data are reported as percent changes from the previous quarter at an annualized rate.

**Figure 9:**
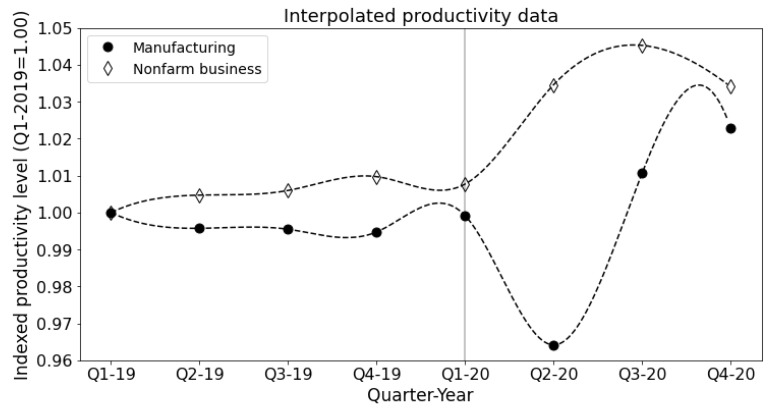
Quarterly labor productivity levels indexed to the Q1-2019 level for the manufacturing (solid dot) and non-farm business (diamond) sectors. Dashed lines show the cubic spline interpolation for each series. The vertical line indicates the beginning of the Covid-19 shock in Q1-2020.

To use these data in our model, we converted the quarterly annualized values into periodic values indexed to the productivity level in Q1-2019. We then interpolated the periodic (quarterly) data to estimate the monthly changes in productivity. We used a common interpolation technique known as cubic spline interpolation, which yields a piece-wise polynomial with two continuous derivatives that necessarily pass through each data point. These properties, among others, make splines a helpful tool to estimate the dynamics in discrete data, such as in Ilyasov [27]. The interpolated BLS data is plotted in Figure 9, where we indicate the onset of the Covid-19 shock.

The productivity data show an interesting distinction between the responses to Covid-19 of the Manufacturing and Non-Farm businesses. Beginning in Q1-2020, each sector experienced significant changes in labor productivity compared with the typical fluctuations of the preceding year. Manufacturing productivity fell roughly −13.3% in Q2-2020, while Non-Farm business rose by 11.1% on an annualized basis. The decrease in Manufacturing productivity captures the drop in output (−46.4%), which outpaced the decrease in hours worked (−38.1%). Global supply chains experienced intense volatility exacerbated by social distancing measures implemented in manufacturing centers that could not be operated remotely.

Non-Farm business, on the other hand, experienced productivity gains due to the hours worked falling more sharply compared to the output (−38.1% and −36.8%). This finding may be due to large amounts of low-wage or part-time workers losing their jobs. Additionally, some industries within Non-Farm business may have had the opportunity to capitalize on the stay-at-home orders and telework technology to increase the hours worked, by, e.g., reducing commuting times, allowing flexible work schedules, and holding meetings with clients and coworkers virtually.

**Figure 10:**
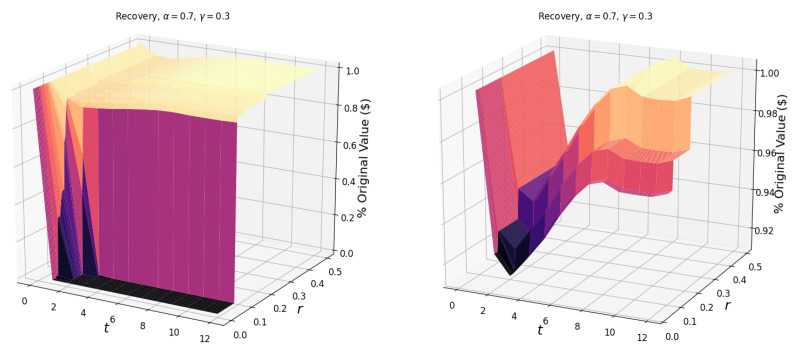
Network response to full 12-month time series of employment levels and the selected productivity measures.

With both the employment and productivity data, our model displayed a strong V-shaped recovery beginning in the third period, corresponding to June 2020 and extending into October 2020 as shown in Figure 10, for the region of no industry failure (r>0.225). The recovery then begins to taper off in November, experiencing a small pullback in the following months. The strong initial recovery is reflected in the employment data, with 13 of the 15 sectors showing increased levels of employment, led by Leisure and Hospitality with a 19.8% month-to-month change, followed by Other Services (7.9%), and Retail Trade (6.3%). The intermediate surface (0.124<r<0.225) corresponds to the failure of the Leisure and Hospitality sector, while the lowest surface ($r_{c}<r<0.124$) includes the failure of Other Services. In the final period, corresponding to March 2021, we see renewed growth as 916,000 jobs are added along with the approval of the $1.9 trillion American Rescue Plan stimulus package, lifted restrictions on stay-at-home orders, and improved vaccine distribution.

**Figure 11:**
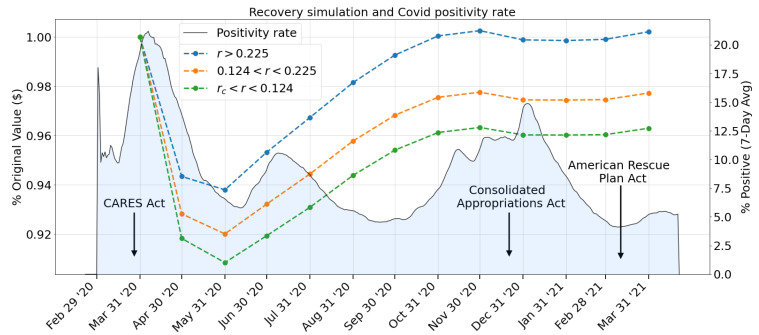
Comparison of the network response (dashed lines, left vertical axis) with the Covid-19 positivity rates in the U.S. (solid line, right vertical axis). The network response is shown as a projection in the (z,t) plane of Figure 10 and is indexed relative to the March 2020 output levels. The dates of signing the three U.S. stimulus packages are indicated: the CARES Act—27 March 2020, Consolidated Appropriations Act—21 December 2020, and American Rescue Plan Act—11 March 2021. Covid-19 positive testing data provided by the Center for Disease Control and Prevention (CDC).

To better understand the context of the recovery surface in Figure 10, we plot the recovery surface projection onto the (% Original Value, t) plane along with the U.S. Covid positivity rate, and the dates of the signing of the three U.S. economic stimulus bills in Figure 11.

On 27 March, 2020, days before the most prominent peak of the measured Covid-19-positivity rate (7-day average of the fraction of positive cases of all reported RT-PCR tests, https://covid.cdc.gov/covid-data-tracker (accessed on 18 April 2021)), the government passed the $2.2 trillion CARES Act in response to the Covid-19 economic fallout. The bill included one-time payments to individuals filing a tax return in the U.S., increased unemployment benefits, the creation of the Paycheck Protection Program (PPP) for small businesses, corporate loans, and aid to local and state governments, all intended to suppress the impact of economic losses in the following months.

The beginning of the modeled recovery period coincides with the lowest positivity rates recorded since data collection began on 1 March 2020. Despite the resurgence of cases in July 2020, the recovery rate remained steady through September 2020 before slowing down and momentarily falling, as the third wave of infections appeared at the end of 2020 to the beginning of 2021. During the slight pullback in network output and the third wave of Covid-19 infections, the U.S. government passed the Consolidated Appropriations Act on 21 December 2020, which included $900 Bn in pandemic relief funds.

The network output remains flat through February 2021 and then shows renewed growth among the third relief bill, the $1.9 trillion American Rescue Plan Act signed on 11 March 2021. Starting in March 2021, we observe positive economic growth, accompanied by the lowest Covid-19 positivity rates, which could signal a continuing recovery of the U.S. economy, especially in light of increased vaccine availability for anyone 16 and older, representing the category that includes the U.S. workforce population.

## 4. Discussion

In this paper, we developed a dynamic cascade model to explore the response of the U.S. inter-industry network to various shocks in production capacity, including single industry disruptions and the reduction in economic output due to the Covid-19 pandemic. Our model makes two primary assumptions, the first being that the resilience and elasticity of labor and input are constant across all industries. The model does allow for these parameters to be tuned for individual industries, provided that one has a method to estimate these parameters in each industry.

Significant deviations from the commonly accepted elasticity values are investigated in the Appendix A (Figure A2a); however, it is well known that production is closely approximated by constant returns to scale function. Secondly, we assume that industries may still produce some fraction of their original output if one or more of their input suppliers has failed.

In reality, some industries may be more important than their monetary value of inputs, e.g., a disruption to the utilities industry may be much more damaging than our model may currently predict. Including the substitutability between various products and industries, as well as accounting for differences in elasticities between industries, is a potential avenue for future improvement of the current model. Our model is limited because there is no contribution of price or wage changes and no possibility of failed industries to re-enter the network.

For single industry shocks, we find that the stability of the network varies with the shock magnitudes and with the industry receiving the initial shock. By ranking the stability levels across all shock magnitudes for each industry, we identify the industries that present the most considerable risk of initiating a catastrophic cascade: Professional Services (PROF), Finance, Insurance, and Real Estate (FIRE), and Manufacturing (31G).

While these are the three largest industries in the network, in addition to the size that determines the damage to the entire network, the industrial interconnections play a significant role. The ranking metric was found to be robust against variations in the two elasticity parameters in decreasing returns to scale (α+γ<1) and constant returns to scale scenarios (α+γ=1). The ranking is unchanged for some increasing returns to scale scenarios (α+γ>1); however, at large enough elasticity values, the ranking differs.

In the Covid-19 disruption, we find that the three most damaging sectors were Manufacturing, Professional Services, and Leisure and Hospitality. While Manufacturing and Professional Services sustained sizable initial shocks, their size in the network primarily dictates their damaging behavior. Leisure and Hospitality, on the other hand, is much smaller than 31G and PROF measured by GDP, yet contributes roughly the same amount to the total output reduction due to its large initial shock.

For regions of *r* in which no industries fail, the Covid-19 shock causes an approximately 9% reduction in the network output after three time periods. For comparison, the U.S. GDP fell at an annualized rate of 31.7% in the second quarter of 2020 as reported by the BEA, which corresponds to a 9% quarterly reduction. The model presented by Luo et al. [22] estimated a 26% decrease in GDP as of 15 April 2020. Makridis and Hartley [23] estimated that two months of partial economic shutdown would cause a 10% reduction in GDP. Both of these studies used the ability of industries to telework as a proxy for labor supply.

In addition to the initial shock, our model can include the effects of variations in productivity or labor supply at any period of the cascade, which allows us to estimate economic recovery scenarios. We find that between March 2020 and March 2021, the period for which we analyzed the effect of Covid-19 on the U.S. economy, there is a sharp initial reduction in the economic output followed by a gradual recovery and reaching the initial pre-Covid economic output levels.

This result is in agreement with the BEA estimate that the U.S. GDP is up 1.5% over the same period (March 2020 to March 2021). Our result is robust, with a variation of less than 1% for a ±10% change in the elasticity parameters. The recovery was supported by $5 trillion in cumulative government stimulus, an adaptation to work-from-home policies where possible, and the rapid development of highly effective mRNA vaccines as well as their increasing availability.

Our model’s policy implications include the possibility of monitoring the economic system in real-time for structural changes. The model can serve as an early warning indicator of economic disruption by assessing the potential damage of an economic shock to an entire financial and economic network. Policymakers and regulators could utilize the model as a monitoring tool and institute appropriate recovery practices that could soften the impact of future economic disruptions.

## Figures and Tables

**Table 1:** Ranked list of average value of $r_{c}$∀β, for each single-industry shock scenario. For example, the entry for manufacturing (31G) is 0.077, which is its average value of $r_{c}$ in Figure 3.

Prof. Services	Fin./Insur./R.E.	Manufacturing	Wholesale Trade	Mining
(PROF)	(FIRE)	(31G)	(42)	(21)
0.085	0.081	0.077	0.049	0.046
**Agric./Forest./Fish**	**Transp./Warehouse**	**Information**	**Government**	**Leisure/Hosp.**
**(11)**	**(48TW)**	**(51)**	**(G)**	**(7)**
0.034	0.032	0.032	0.023	0.019
**Retail Trade**	**Construction**	**Utilities**	**Other Services**	**Educ./Health**
**(44RT)**	**(23)**	**(22)**	**(81)**	**(6)**
0.018	0.016	0.012	0.010	0.004

**Table 2:** Percent change in employment for the 15 sector level industries between March and April 2020.

Agric./Forest./Fish.	Mining	Utilities	Construction	Manufacturing
(11)	(21)	(22)	(23)	(31G)
−1.3%	−7.4%	−0.66%	−13.4%	−10.3%
**Wholesale Trade**	**Retail Trade**	**Transp./Warehouse**	**Information**	**Fin./Insur./R.E.**
**(42)**	**(44RT)**	**(48TW)**	**(51)**	**(FIRE)**
−6.5%	−14.7%	−9.9%	−9.7%	−3.0%
**Prof. Services**	**Educ./Health**	**Leisure/Hosp.**	**Other services**	**Government**
**(PROF)**	**(6)**	**(7)**	**(81)**	**(G)**
−10.3%	−10.7%	−47.0%	−21.9%	−4.2%

**Figure A1:**
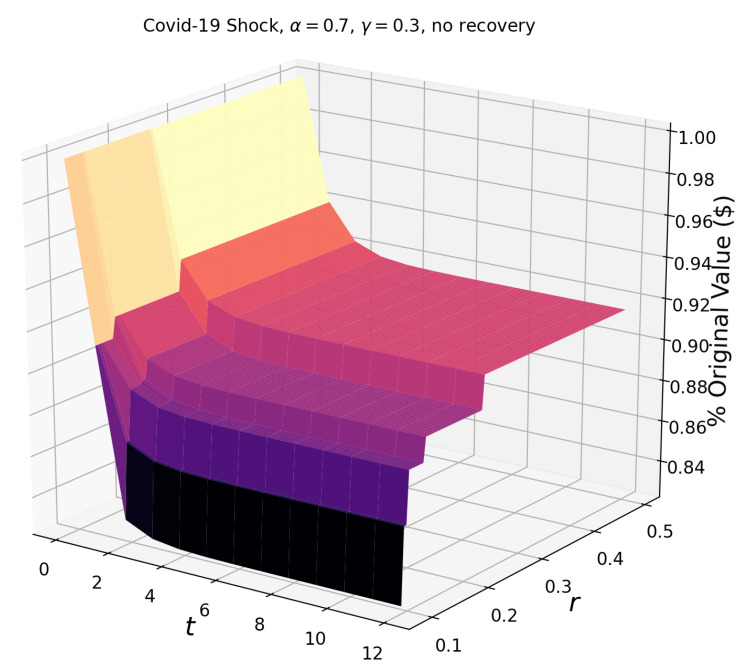
Response of the network to the initial Covid-19 labor shock, for $r>r_{c}$.

## Data Availability

Data available in publicly accessible repositories that do not issue DOIs. BEA Total Requirements Table [https://apps.bea.gov/iTable/itable.cfm?reqid=52&step=1] (accessed on 18 April 2021); BLS Employment by Industry [https://www.bls.gov/charts/employment-situation/employment-levels-by-industry.htm] (accessed on 18 April 2021); BLS Unemployment Rate [https://data.bls.gov/cgi-bin/surveymost] (accessed on 18 April 2021); BLS Major Sector Productivity [https://data.bls.gov/cgi-bin/surveymost?pr] (accessed on 18 April 2021); CDC Covid Data Tracker [https://covid.cdc.gov/covid-data-tracker/#datatracker-home] (accessed on 18 April 2021).

## Abbreviations

The following abbreviations are used in this manuscript:

BEA	Bureau of Economic Analysis
BLS	Bureau of Labor Statistics
CDC	Center for Disease Control and Prevention
GDP	Gross Domestic Product
